# Exploring the Link Between Stress-Induced Naive T-Cells and Esophageal Squamous Cell Carcinoma Risk: A Multi-Omics Investigation

**DOI:** 10.1245/s10434-025-18541-w

**Published:** 2025-11-04

**Authors:** Jingge Cheng, Dengfeng Zhang, Longyu Zhu, Huihai Xu, Hongye Zhao, Ping Zhu, Yishuai Li

**Affiliations:** 1 The Fourth Hospital of Hebei Medical University, Shijiazhuang, Hebei China; 2 Guangdong Provincial People’s Hospital, Southern Medical University, Guangzhou, Guangdong China; 3 Hebei Chest Hospital, Hebei Provincial Key Laboratory of Pulmonary Diseases, Shijiazhuang, Hebei China

**Keywords:** Single-cell technologies, Mendelian randomization, Esophageal squamous cell carcinoma, Naive T cells, Heat shock proteins

## Abstract

**Background:**

The dynamic crosstalk between immunoregulatory constituents and neoplastic evolution underscores the centrality of immune homeostasis in oncogenesis. naïve T-lymphocytes, as primary architects of adaptive immunity, are critically implicated in antitumor responses. Deciphering their mechanistic linkage to esophageal carcinogenesis is vital for elucidating immune-mediated susceptibility and malignant progression. This investigation synergizes Mendelian randomization with single-cell multi-omics to dissect this pathobiological nexus.

**Methods:**

A bidirectional two-sample Mendelian randomization framework was deployed to infer causal associations between leukocyte subsets and esophageal squamous cell carcinoma. Genome-wide summary statistics were analyzed from 476,306 malignancy cases and immunophenotypic data spanning 731 European-ancestry participants. Complementary single-cell transcriptional profiling of tumor-adjacent dyads provided mechanistic validation.

**Results:**

Inverse variance-weighted regression demonstrated a positive causal effect of naive T-cell abundance on esophageal cancer risk (odds ratio 1.14; 95% confidence interval 1.03–1.27; *P* = 0.021). Single-cell analyses unveiled tumor-infiltrating naïve T-lymphocytes in a metabolically quiescent state with suppressed effector differentiation trajectories, indicative of microenvironment-driven anergy. This functional impairment correlated with compromised immunoediting capacity, potentiating neoplastic immune evasion mechanisms.

**Conclusions:**

Our integrative analysis establishes naïve T-cell abundance as a novel causal risk factor for esophageal squamous cell carcinoma, mediated through metabolic silencing and differentiation blockade within the tumor niche. These findings redefine the immunopathogenic paradigm of esophageal carcinogenesis, suggesting that therapeutic strategies targeting T-cell metabolic reprogramming may disrupt immune-evasion pathways. The demonstrated synergy between population-level genetic inference and single-cell mechanistic validation provides a transformative framework for cancer immunology research.

**Supplementary Information:**

The online version contains supplementary material available at 10.1245/s10434-025-18541-w.

Esophageal cancer is a major global health issue, with particularly high incidence rates in developing countries.^1,2^ Esophageal squamous cell carcinoma (ESCC) accounts for approximately 85% of esophageal cancer cases worldwide, with a disproportionately high burden in regions such as East Asia and East Africa.^3,4^ Despite recent advancements in treatment, the management of ESCC remains challenging and is often associated with poor patient outcomes.^5^ The immune system plays a critical role in both the prevention and the progression of cancer, and dysregulation of immune cell functions can lead to immune evasion and tumor development.^6,7^ Among the immune cells, naïve T-cells are a crucial subset involved in the initiation and regulation of immune responses.^8,9^ These cells typically function to recognize and eliminate abnormal cells, thereby maintaining immune homeostasis. However, recent studies suggest that in the tumor microenvironment, naïve T-cells may undergo stress-induced functional changes that promote tumor progression and immune escape.^10–12^ Within the tumor microenvironment, naïve T-cells may be subjected to stress and pressure, resulting in alterations in their function and phenotype, thereby facilitating tumor escape and progression.^13,14^

The potential involvement of naïve T-cells in ESCC highlights a critical research gap that requires further exploration. Given that cellular stress is a hallmark of the tumor microenvironment, and heat shock proteins (HSPs) are key mediators of the cellular stress response, we hypothesized that HSPs might be involved in the stress-induced alterations of naive T cells. Addressing this gap is essential for understanding the role of immune cells in cancer risk and progression. To investigate this, advanced techniques such as Mendelian randomization (MR) and single-cell sequencing have been employed. MR is a powerful method that leverages genetic variants to assess causal relationships, providing more objective and reliable results in understanding disease mechanisms.^15,16^ Single-cell sequencing, on the other hand, allows for a detailed analysis of gene expression and immune cell behavior at the individual cell level, particularly within the complex and heterogeneous tumor microenvironment.^17,18^ Furthermore, heat shock proteins (HSPs) are a class of proteins whose expression increases under cellular stress conditions,^19^ exerting protective effects^20^ and promoting cellular recovery.^21^ For instance, HSP70 is abundantly expressed on the surface of tumor cells.^22^ These HSP70-positive tumor cells actively release exosomes with surface-bound HSP70, which subsequently stimulates natural killer (NK) cells.^23^ Multhoff et al.^22^ demonstrated that NK cells, pre-stimulated with HSP70 protein or the 14-mer HSP70 peptide (TKD) in the presence of interleukin (IL)-2 or -15, can recognize these surface-bound HSP70 molecules.^24,25^ In the tumor microenvironment, HSPs may impact the function and phenotype of immune cells, consequently influencing the immune response and therapeutic outcomes.^26–28^ Therefore, in-depth exploration of the effects of stress-induced responses on naïve T-cells and the role of HSPs therein holds significant relevance for understanding the onset and progression of ESCC.

In this study, we aim to address the research gap by investigating the causal relationship between naïve T-cells and ESCC risk using MR. Additionally, single-cell sequencing will be utilized to validate and further explore the role of naïve T-cells within the tumor microenvironment. By integrating these cutting-edge technologies, our goal is to elucidate the mechanisms through which naïve T-cells contribute to ESCC progression. This study seeks to provide a foundation for the development of novel immunotherapeutic strategies targeting naïve T-cells in ESCC.

## Methods

### Immunity-Wide and Esophageal Cancer Genome-Wise Association Study Data

Summary statistics for each immunophenotype are publicly accessible through the genome-wide association study (GWAS) catalog, with accession numbers ranging from GCST90001391 to GCST90002121, covering a comprehensive set of 731 immunophenotypes. This encompassing dataset includes absolute cell counts (n=118), median fluorescence intensity reflecting surface antigen levels (n=389), morphological parameters (n=32), and relative cell counts (n=192). Encompassing various developmental stages and cell types of immune cells, these features were derived from the original GWAS conducted on 3757 European individuals, utilizing non-overlapping cohorts. The significance level for instrumental variables (IV) in each immunophenotype was conservatively set at 1 × 10^(-5). To ensure the robustness of our analyses, we pruned the single nucleotide polymorphisms (SNPs) using a linkage disequilibrium threshold of <0.1 within a 500 kb distance. This comprehensive dataset, derived from a diverse range of immunophenotypes and meticulously curated through stringent analyses, serves as a valuable resource for understanding the genetic determinants of immune cell characteristics. The stringent significance threshold and SNP pruning contribute to the reliability and precision of our results, providing a solid foundation for further investigations into the intricate relationships between genetic variants and immune cell traits. ESCC (n = 476,306) GWAS data were obtained from the GWAS catalog (accession numbers ebi-a-GCST90018841).

### Single-Cell RNA-Seq Analysis

The scRNA-seq data of human patients with ESCC were downloaded from the GEO database with the accession number: GSE196756. We employed the Seurat R package for the identification of distinct cell types and exploration of variations in immune cell infiltration. Cells failing to meet specific criteria, such as those with fewer than 200 genes, over 5000 genes, or exhibiting >20% mitochondrial expression, were excluded from the analysis. Raw counts underwent normalization using the ‘NormalizeData’ function, and variable genes were identified through the ‘FindVariableGenes’ function. Subsequently, dataset expression values underwent scaling and centering using the ‘ScaleData’ function to reduce dimensionality. Principal component analysis and the uniform manifold approximation and projection methods were utilized for data visualization in lower dimensions, with the first two dimensions selected for plotting. Cell clustering was executed using the ‘FindClusters’ function, and highly expressed genes within each cell cluster were identified via the ‘FindAllMarkers’ function. Additionally, the ‘FindMarkers’ function was employed to identify differentially expressed genes (DEGs) between two cell populations. This comprehensive workflow ensured a rigorous analysis of single-cell data, facilitating the identification of distinct cell types and providing insights into immune cell heterogeneity within the studied context.

### Pseudo-Time Analysis

Monocle3 was employed to investigate the developmental timing and trajectory of T-cells. This algorithmic tool identifies the sequential gene expression changes that occur within each cell during a dynamic biological process. Using unsupervised or semi-supervised learning, Monocle constructs a trajectory that arranges cells in a simulated time sequence based on the data.

### Mendelian Randomization

MR analysis has been widely recognized as a complementary method to randomized controlled trials.^29^ This approach primarily uses genetic variations as IVs to simulate and examine the causal relationship between exposure factors and diseases.^30^ A key characteristic of genetic variants that enhances their potential as powerful IVs is their random allocation during gamete formation and conception, according to Mendel’s laws. While alleles are technically randomly allocated within families,^31,32^ their distribution across a population is approximately random.^33,34^ Consequently, genetic variants serve as instruments for both randomization and causal effect estimation.

In addressing potential challenges such as pleiotropy or weak instrument bias, we adopted several robust approaches, including inverse variance weighting (IVW), MR Egger, weighted median (WM), penalized WM, robust-adjusted profile score (MR RAPS), and MR LASSO. IVW served as the primary analysis, employing an inverse-variance weighted formula to estimate combined causal effects while simultaneously minimizing the variance of the weighted average. MR Egger, assuming independence of pleiotropic associations, performed a weighted linear regression of outcome coefficients on exposure coefficients. The WM estimator demonstrated comparable efficiency to the IVW method, serving as a supplementary approach. Penalized WM and MR LASSO were employed in cases of IV heterogeneity, providing robustness. These diverse methodologies collectively enhance the reliability and robustness of our MR analysis, accommodating various scenarios and potential biases. The utilization of these approaches aligns with best practices in the field and contributes to the credibility of our findings in accordance with the standards expected in scientific publications. MR Egger analysis was conducted to assess potential pleiotropic effects based on the intercept, where intercepts close to zero indicate the absence of horizontal pleiotropy. Furthermore, we employed the MR-PRESSO test to identify outliers indicative of horizontal pleiotropy in multi-instrument summary-level MR. The global test associated with MR-PRESSO indicated the presence or absence of pleiotropy. The heterogeneities across analyses were quantified using the Cochran Q statistic for both IVW and MR Egger methods. To address potential outliers and reduce heterogeneity, we employed MR-PRESSO and radial regression. These methods identify and eliminate outliers, contributing to a more robust and reliable analysis. Additionally, a "leave-one-out" sensitivity analysis was applied, wherein MR was performed iteratively, each time leaving out a single SNP to identify potentially influential SNPs. These comprehensive analytical approaches contribute to the validity and robustness of our MR analysis, ensuring the consideration and mitigation of potential sources of bias such as pleiotropy and outliers. The stringent methodology employed enhances the reliability of our findings, aligning with the rigorous standards expected in scientific publications.

### Immunohistochemistry and Immunofluorescence

The tissue samples utilized for immunohistochemical and immunofluorescence analyses were obtained from paired tumor and adjacent normal tissues with ESCC. All specimens were collected during surgical resection at the Hebei Chest Hospital (Hebei Province, China), followed by formalin fixation and paraffin embedding for subsequent sectioning and staining procedures. We performed immunohistochemical and immunofluorescence staining on paired tumor and adjacent normal tissues from three patients with ESCC. For the immunohistochemical staining: 4-μm sections from paraffin-embedded tissues were deparaffinized in xylene and rehydrated through graded ethanol. After antigen retrieval (method if performed), sections were blocked with 10% normal goat serum (710027, KPL, USA) for 1 h at room temperature. The sections were then incubated with primary antibody against DNAJB1 (82676-1-RR, proteintech; 1:1000) overnight at 4°C. After washing, HRP-conjugated rabbit immunoglobulin secondary antibody (021516, KPL, USA) was applied for 1 h at room temperature. Signal was developed using DAB substrate, followed by hematoxylin counterstaining. Slides were dehydrated, cleared, and mounted for evaluation. For multiplex immunofluorescence staining, rehydrated tissue sections were subjected to heat-induced antigen retrieval in citrate buffer (pH 6.0) at 95°C for 15 min. After cooling and phosphate-buffered saline (PBS) washes, sections were blocked with 5% bovine serum albumin in PBS for 1 h at room temperature. The sections were then incubated with a primary antibody cocktail containing rabbit anti-DNAJB1 (82676-1-RR, proteintech; 1:1000), goat anti-CCR7 (MBS626366, MyBioSource; 1:500), and mouse anti-CD3 (60181-1-Ig, proteintech; 1:800) overnight at 4°C in a humidified chamber. After three washes with PBS, the sections were incubated with species-specific secondary antibodies for 1 h at room temperature protected from light. Nuclei were counterstained with DAPI (1 μg/mL; D1306, Invitrogen) for 5 min. Slides were mounted with ProLong Gold Antifade Mountant (P36930, Invitrogen) and stored at 4°C in the dark until imaging.

### Statistical Methods

All data computations and statistical analyses were conducted using R software (https://www.r-project.org/, version 4.1.2). For comparisons between two groups of continuous variables, statistical significance for normally distributed variables was assessed using independent Student’s t-test, while differences among non-normally distributed variables were analyzed using the Mann–Whitney U test (also known as the Wilcoxon rank-sum test). Unless otherwise specified, Spearman correlation analysis was employed to calculate correlation coefficients between different molecules, with all statistical P-values being two-sided. A P-value <0.05 was considered statistically significant.

## Results

### Exploring the Causal Effects Between Immune Cells and ESCC

**Fig. 1:**
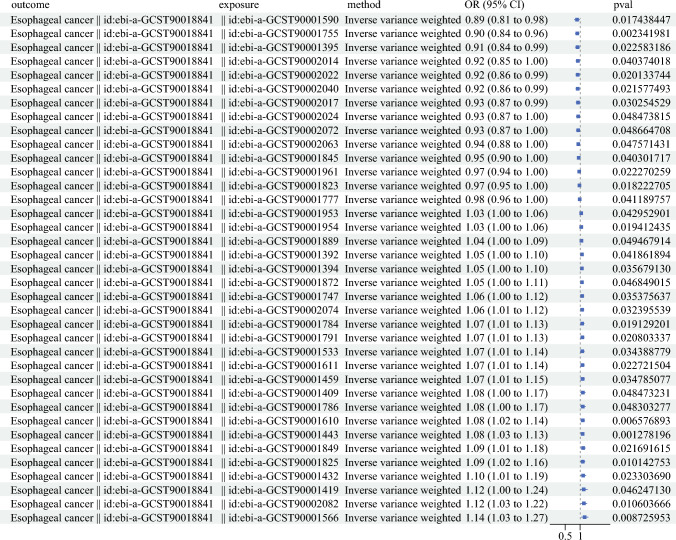
Univariate Mendelian randomization analysis revealing causal relationships between immune cell phenotypes and esophageal squamous cell carcinoma (ESCC)

We investigated the causal association between the phenotype of immune cells and ESCC. The IVW method was primarily employed for our analysis. Our findings consistently demonstrate that various immune cell phenotypes are causally linked to ESCC. Notably, ebi-a-GCST90001566 (naïve CD4-CD8- T cell absolute count) exhibits a significant causal relationship with ESCC, boasting a maximum odds ratio (OR) of 1.14, a 95% confidence interval (CI) ranging from 1.03 to 1.27, and a P-value <0.05 (Fig. 1).

**Fig. 2:**
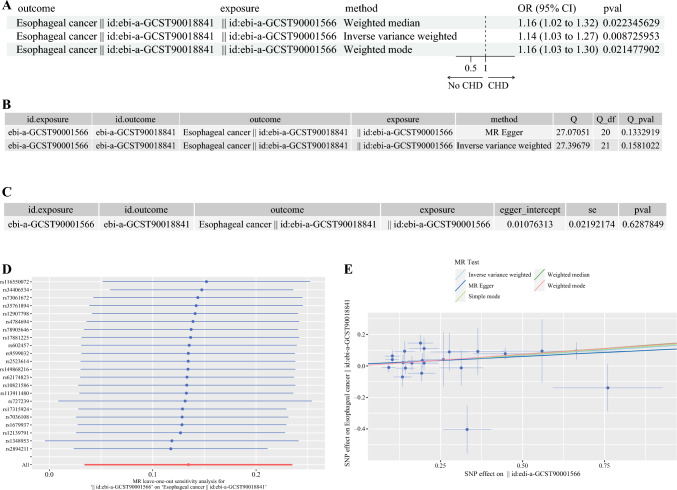
Three additional methods, namely weighted median, inverse variance weighted, and weighted mode, used to validate our analytical results. **A** Univariate Mendelian randomization (MR) analysis revealing causal relationships between naïve T-cells and esophageal squamous cell carcinoma (ESCC). **B** Heterogeneity test for the causal relationship between naïve T-cells and ESCC. **C** Horizontal pleiotropy test for the causal relationship between naïve T-cells and ESCC. **D** The leave-one-out analysis consistently demonstrated trends across all included single nucleotide proteins (SNPs). **E** Scatterplot demonstrating the reliability of results across multiple methods. CHD, ; CI, confidence interval; OR, odds ratio; pval, p-value

Furthermore, we employed three additional methods (WM, IVW, and weighted mode), to validate our analytical results. The outcomes consistently indicate a causal relationship between naïve CD4-CD8- T-cell absolute count and ESCC using all three approaches (Fig. 2A). To assess heterogeneity in our results, we performed tests using MR Egger and IVW methods, both yielding p-values >0.05. This suggests the absence of heterogeneity in our analysis results. A thorough examination for horizontal pleiotropy using the MR Egger method revealed a p-value of 0.62 for the MR Egger regression intercept, surpassing the threshold of 0.05. This indicates the absence of horizontal pleiotropy in our analysis results (Fig. 2B,C). The leave-one-out (LOO) analysis consistently demonstrated trends across all included SNPs in our study, further corroborating the robustness of our findings. Scatter plots provided visual evidence of the stability and reliability of our results (Fig. 2D,E). To address this, we conducted a reverse MR analysis. The results from this validation demonstrate a p-value >0.05, indicating that there is no reverse causality between naïve T-cells and ESCC (Table S1). This supports the robustness of our initial findings.

### Exploring the Infiltration Dynamics of T-Cells in ESCC

**Fig. 3:**
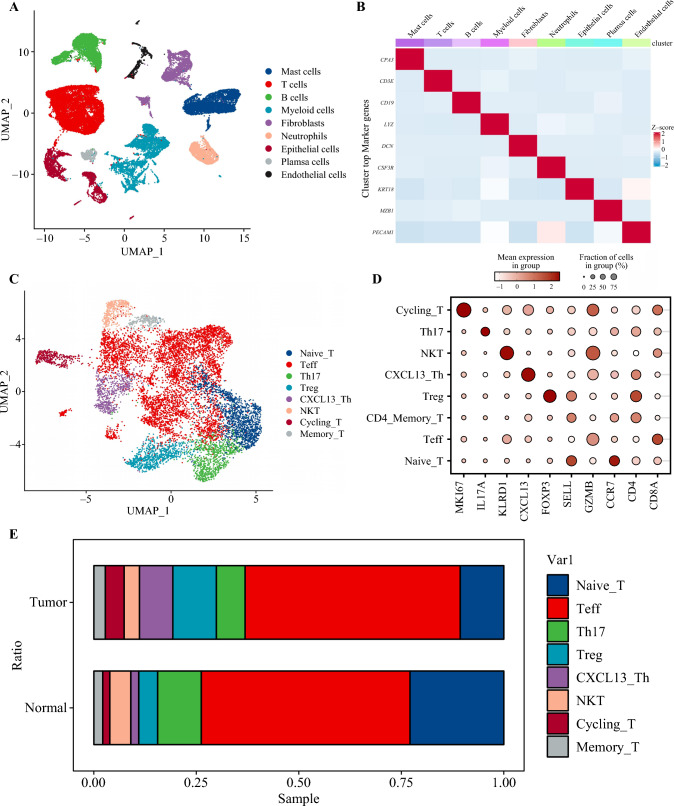
Exploring the infiltration dynamics of T-cells in esophageal squamous cell carcinoma (ESCC). **A** uniform manifold approximation and projection (UMAP) plot illustrating distinct cell types. **B** Expression profile of cell marker genes. **C** UMAP plot depicting different T-cell subtypes. **D** Expression profile of T-cell marker genes. **E** Bar graph illustrating infiltration levels of different T-cell types in normal and tumor tissues. NKT, natural killer T-cells; Th17, T-helper cell type 17; Treg, regulatory T-cells

To further investigate T-cell infiltration in normal esophageal tissues compared with in ESCC, we reanalyzed single-cell data from six patients: three with ESCC and three with normal esophageal tissues. Utilizing cell marker genes such as CPA3, CD3E, CD19, LYZ, DCN, CSF3R, KRT18, MZB1, and PECAM1, we classified cells into distinct populations, including mast cells, T-cells, B-cells, myeloid cells, fibroblasts, neutrophils, epithelial cells, plasma cells, and endothelial cells. We employed uniform manifold approximation and projection for dimensionality reduction and visualization of the data (Fig. 3A,B).

Furthermore, we conducted a reductive clustering analysis specifically focusing on T-cells, categorizing them into distinct subtypes based on their functional states. The T-cell subtypes included proliferating T-cells (Cycling_T, marked by Mki67), t-helper (Th)-17 cells (IL-17A-expressing), NK T-cells (KLRD1-expressing), CXCL13Th cells (CXCL13-expressing), regulatory T-cells (Treg, marked by FOXP3), CD4-positive memory T-cells (CD4_Memory_T, marked by SELL), effector T-cells (Teff, marked by GZMB), and naïve T-cells (naïve T, marked by CCR7) (Fig. 3C,D).

Comparing tumor tissues with normal esophageal tissues, we observed a significant increase in Treg-cell infiltration and a notable decrease in naïve T-cell infiltration in the tumor tissues (Fig. 3E). These findings suggest a potential intricate relationship between naïve T-cells and the occurrence and progression of ESCC. The elevated presence of Treg cells in tumor tissues may indicate a role in immune regulation within the tumor microenvironment, while the decreased infiltration of naïve T-cells raises intriguing questions about their potential involvement in the pathogenesis of ESCC.

### Exploring Changes in Naïve T-Cells in ESCC

**Fig. 4:**
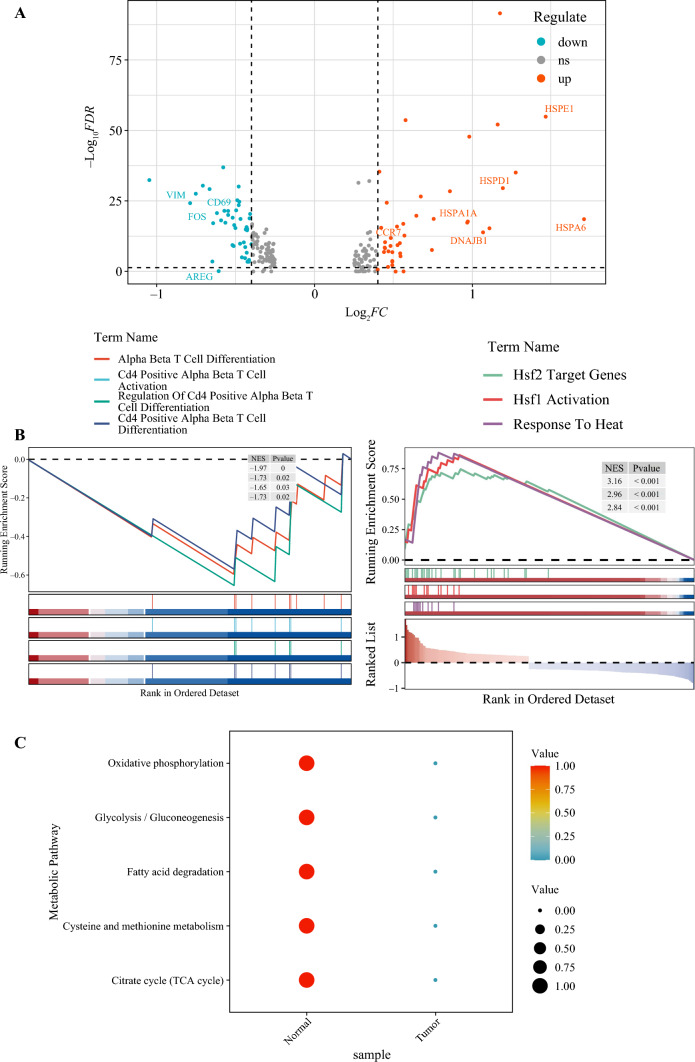
Exploring changes in naïve T-cells in esophageal squamous cell carcinoma (ESCC). **(A)** Differential gene expression analysis revealing variances in naïve T-cells between tumor and normal tissues. **B** gene set enrichment analysis depicting the enrichment levels of multiple pathways in naïve T-cells from tumor and normal tissues. **C** Bubble chart illustrating the enrichment levels of multiple metabolic pathways

To explore the changes in naïve T-cells within tumor tissues, we examined the DEGs associated with naïve T-cells in tumor tissues compared with normal tissues. The elevated expression of HSPs such as HSPD1, HSPE1, HSPA1A, HSPA6, and DNAJB1 suggests that naïve T-cells in tumor tissues may be under a state of stress or heat shock (Fig. 4A).

Furthermore, in tumor tissues, naïve T-cells exhibited higher expression levels of CCR7, whereas, in normal tissues, naïve T-cells displayed increased CD69 expression. This observation implies that naïve T-cells in tumor tissues may adopt a more immature or naïve state, characterized by heightened CCR7 expression, which is indicative of their potential for migration, and lower CD69 expression (Fig. 4A), suggesting a less activated or mature status than naïve T-cells in normal tissues. These findings provide valuable insights into the nuanced alterations in naïve T-cells within the tumor microenvironment, shedding light on their potential role in the context of ESCC.

We performed gene set enrichment analysis on the DEGs of naïve T-cells in tumor tissues compared with normal tissues. Our analysis revealed a significant downregulation of pathways related to T-cell differentiation and activation, including alpha beta T-cell differentiation, Cd4 positive alpha beta T-cell activation, regulation of Cd4 positive alpha beta T-cell differentiation, and Cd4-positive alpha beta T-cell differentiation (Fig. 4B).

Moreover, pathways associated with stress response, such as Hsf2 target genes, Hsf1 activation, and response to heat, also exhibited significant downregulation in tumor tissues. These findings suggest that naïve T-cells in tumor tissues are characterized by a diminished capacity for differentiation and activation in pathways crucial for T-cell function. The observed downregulation of stress response pathways further supports the notion that naïve T-cells within the tumor microenvironment may indeed be in a state of reduced functional capability and heightened stress (Fig. 4B).

Furthermore, we conducted an analysis of metabolism-related pathways in naïve T-cells from tumor tissues and normal tissues. The results revealed that naïve T-cells in tumor tissues exhibit lower levels of several metabolic pathways, including oxidative phosphorylation, glycolysis/gluconeogenesis, fatty acid degradation, cysteine and methionine metabolism, and the citrate cycle (Fig. 4C). These findings suggest that naïve T-cells within the tumor microenvironment may experience metabolic alterations, potentially indicative of a reduced energy production capacity and altered metabolic priorities. The downregulation of these key metabolic pathways in naïve T-cells further underscores the complex interplay between the tumor microenvironment and the metabolic reprogramming of immune cells, contributing to our understanding of the functional adaptations of naïve T-cells in the context of ESCC.

### Weighted Gene Co-Expression Network Analysis of Gene Modules Regulated by Naïve T-Cells

**Fig. 5:**
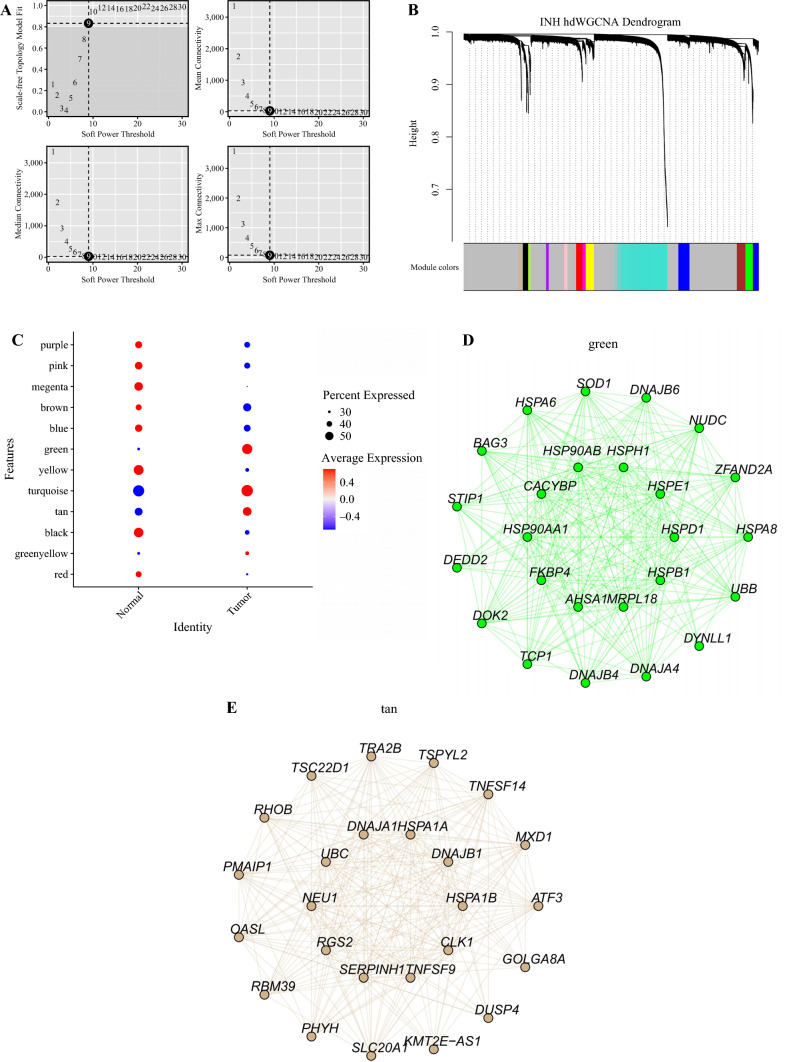
Weighted gene co-expression network analysis (WGCNA) of gene modules regulated by naïve T-cells. **A** Evaluation of the scale-free fit index for different soft-thresholding powers. **B** Hierarchical clustering tree representing co-expressed gene modules. **C** Dot plot illustrating the expression of module genes in tumor and normal tissues. **D** Identification of hub genes in the green modules. **E** Identification of hub genes in the tan modules

To construct a gene co-expression network for naïve T-cells and identify potential regulators influencing naïve T-cell behavior within tumor tissues, we performed weighted gene co-expression network analysis (WGCNA) on the top 2000 highly variable genes in naïve T-cells. The selection of the soft threshold power is a crucial step in building the WGCNA. We conducted a network topology analysis for threshold powers ranging from 1 to 30, ensuring a balance between scale independence and average connectivity (Fig. 5A). We chose a power of 9 as the minimum power, with a scale-free topology fitting index of 0.9 or higher, leading to the generation of a hierarchical clustering tree for the 2000 genes. Ultimately, we identified 12 gene modules in the analysis (Fig. 5B), with the tan, green, and turquoise modules exhibiting higher expression in naïve T-cells from tumor tissues (Fig. 5C). Hub genes were selected for visualization from the green and tan modules, revealing a notable presence of HSP-related genes in both modules (Fig. 5D,E). This observation further supports the notion that naïve T-cells in tumor tissues are regulated by genes associated with HSPs. The identification of these modules and hub genes provides a foundation for understanding the regulatory networks influencing naïve T-cells in the tumor microenvironment.

### Pseudo-Time Reveals Naïve T-Cell Transition Trajectory

**Fig. 6:**
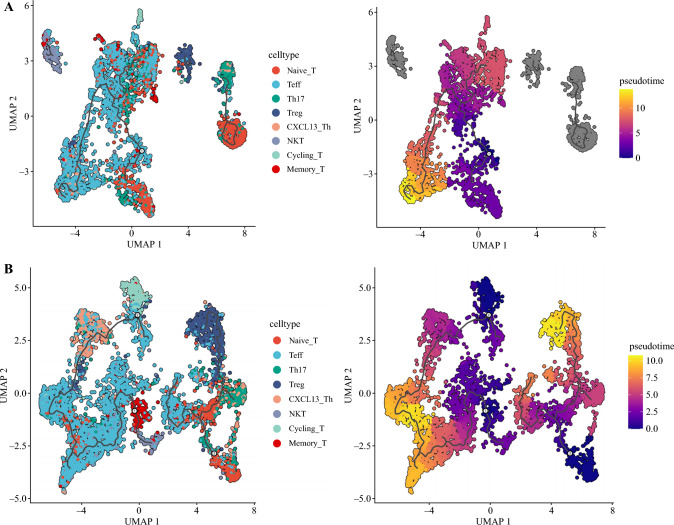
Pseudo-time reveals naïve T-cell transition trajectories. **A** Pseudotime analysis of naïve T-cells in normal tissues. **B** Pseudotime analysis of naïve T-cells in tumor tissues. NKT, natural killer T-cells; Th17, T-helper cell type 17; Treg, regulatory T-cells; UMAP, uniform manifold approximation and projection

To further investigate whether there are differences in the developmental trajectories of naïve T-cells in tumor tissues compared with normal tissues, we employed monocle3 to conduct pseudotime analysis separately for naïve T-cells from tumor and normal tissues. The results revealed distinctive patterns in the developmental trajectories of naïve T-cells between these two tissue types. In normal tissues, naïve T-cells tend to differentiate towards effector T-cells. Conversely, in tumor tissues, naïve T-cells exhibit a preference for differentiation towards Tregs (Fig. 6A,B). This observation suggests that the microenvironment of tumor tissues may influence the fate and differentiation of naïve T-cells, potentially contributing to the altered immune response associated with ESCC. The pseudotime analysis provides valuable insights into the dynamic processes underlying the differentiation of naïve T-cells and their potential role in the context of tumor immune microenvironment.

Finally, through pseudotime analysis, we unveiled distinct developmental trajectories for naïve T-cells in normal and tumor tissues. In normal tissues, naïve T-cells tend to differentiate towards effector T-cells. Conversely, in tumor tissues, naïve T-cells exhibit a preference for differentiating into Tregs. These collective findings underscore the impact of the tumor microenvironment on naïve T-cell dynamics. The observed stress-induced state of naïve T-cells in tumor tissues, marked by weakened differentiation and activation capabilities, coupled with the downregulation of various metabolic pathways, contributes to a diminished capacity for transitioning into effector T-cells. Additionally, the demonstrated preference for differentiating into Tregs in the tumor microenvironment further facilitates immune escape mechanisms within the tumor. Taken together, these results provide a comprehensive view of the altered functional and developmental landscape of naïve T-cells in the context of ESCC. The stress-induced phenotypic changes and the propensity for Treg differentiation collectively contribute to the promotion of immune evasion within the tumor microenvironment.

### Immunohistochemistry and Immunofluorescence Validation

**Fig. 7:**
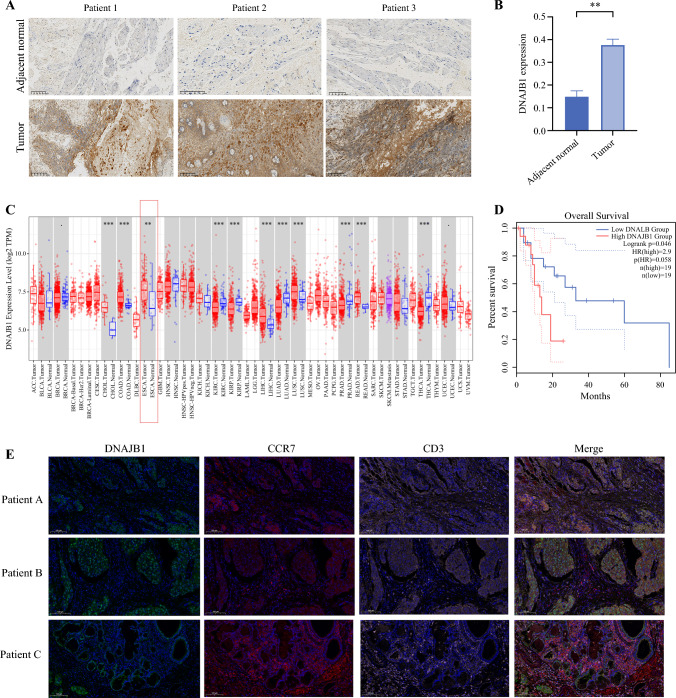
DNAJB1 expression and prognostic correlation in esophageal squamous cell carcinoma (ESCC). **A** Representative immunohistochemistry (IHC) staining images depicting DNAJB1 protein expression in human ESCC and adjacent normal tissue. **B** Statistical analysis of IHC expression levels in tumor tissue compared with adjacent normal tissue. **C** Pan-cancer expression profile of DNAJB1 as analyzed by TIMER. **D** Overall survival curves for patients with ESCC based on data from the GEPIA database. **E** Immunofluorescence staining confirming the relationship between DNAJB1 expression and infiltration of naive T-cells in ESCC specimens. CD3 was utilized as a T-cell marker, whereas CCR7 was used as a marker for naive T-cells. The staining involved DNAJB1 (green), CCR7 (red), CD3 (pink), and DAPI (blue) for nuclear staining. Scale bar: 100 μm. (*p<0.05; **p<0.01; ***p<0.001). HR, hazard ratio

Following our initial investigations, we proceeded to validate functionality using human ESCC tissue. Consistently, we observed a significant upregulation in DNAJB1 protein expression within human ESCC tissues (Fig. 7A) and the statistical results of the immunohistochemical experiments (Fig. 7B). Subsequently, leveraging external databases, we confirmed that DNAJB1 expression in ESCC markedly surpassed that in normal tissue (Fig. 7C). Moreover, analysis using the GEPIA database revealed a negative correlation between DNAJB1 protein expression in ESCC tissues and overall survival (Fig. 7D). Building upon our previous research, which included exploratory analysis utilizing single-cell data from esophageal carcinoma, we elucidated the regulatory influence of DNAJB1 on naive T cells, indicating a positive correlation. To substantiate this regulatory nexus, we employed immunofluorescence techniques to assess DNAJB1 expression alongside CD3, a T-cell marker, and CCR7, a naive T-cell marker. Through immunofluorescence experiments, we demonstrated co-localization of DNAJB1 and CCR7 in naive T-cells (Fig. 7E). This suggests that high expression of DNAJB1 in naive T-cells within the tumor microenvironment induces stress, thereby influencing the differentiation and function of naive T-cells. This further validates our previous analysis results.

In ESCC, the role of stressed naïve T-cells can be understood through several mechanisms revealed by our research. We observed significant functional and state changes in naïve T-cells within the tumor microenvironment, which likely contribute to the development and progression of ESCC. First, our study showed that naïve T-cells in tumor tissues exhibit increased expression of HSPs, including HSPD1, HSPE1, HSPA1A, HSPA6, and DNAJB1. This suggests that the tumor microenvironment induces a stress response in naïve T-cells, potentially placing them in a more "primitive" or stressed state than those in normal tissues. Specifically, naïve T-cells in tumors exhibited higher CCR7 expression and lower CD69 expression. This indicates that tumor-associated naïve T-cells may adopt a more migratory phenotype with reduced activation or maturation than their counterparts in normal tissues. Second, gene set enrichment analysis revealed a downregulation of pathways related to T-cell differentiation and activation in naïve T-cells from tumor tissues. This suggests that the stress-induced state of naïve T-cells in the tumor microenvironment impairs their ability to differentiate and activate properly. Additionally, we observed significant downregulation of stress response pathways in these cells, further supporting the notion that naïve T-cells in the tumor environment are under stress and have a reduced functional capacity. Moreover, metabolism-related pathways in naïve T-cells from tumor tissues were also downregulated, indicating potential metabolic alterations. These changes may reflect a reduced energy production capacity and altered metabolic priorities, contributing to the overall functional adaptations of naïve T-cells within the tumor microenvironment. Overall, the stress-induced state of naïve T-cells, characterized by impaired differentiation and activation, coupled with metabolic changes, highlights their diminished capacity to transition into effector T-cells. Instead, these cells show a preference for differentiating into Tregs within the tumor environment. This shift may facilitate immune evasion mechanisms and contribute to the progression of ESCC. Thus, understanding these altered functional and developmental dynamics of naïve T-cells provides valuable insights into the mechanisms underlying immune evasion and cancer progression in ESCC.

## Conclusion

In this study, we employed a novel integration of MR and single-cell transcriptome sequencing to investigate the causal relationships between immune cell-mediated processes and ESCC. Our findings reveal a significant causal association between naïve T-cell infiltration and ESCC, as confirmed through MR analysis. Furthermore, single-cell sequencing results highlighted that naïve T-cells in tumor tissues exhibit reduced maturation and increased stress responses than those in normal tissues.

The elevated expression of HSPs in naïve T-cells within the tumor microenvironment suggests that these cells may be under stress, which could contribute to their impaired differentiation and activation capabilities. Additionally, the observed downregulation of key metabolic pathways in naïve T-cells further underscores the complex interplay between cellular metabolism and immune function in cancer progression. Our weighted gene co-expression network analysis identified gene modules associated with stress response and HSPs, providing insights into the regulatory networks influencing naïve T-cell behavior in tumor tissues. The pseudotime analysis revealed that naïve T-cells in the tumor microenvironment preferentially differentiate into Tregs, which may facilitate immune evasion. In summary, our study provides a comprehensive view of how naïve T-cells, through stress-induced states and altered differentiation trajectories, contribute to the risk and progression of ESCC. These insights enhance our understanding of the immune landscape within the tumor microenvironment and highlight potential avenues for targeted therapeutic interventions aimed at mitigating the impact of stress-induced alterations in naïve T-cells.

## Discussion

To the best of our knowledge, this study represents the first integration of MR and single-cell transcriptome sequencing analysis to investigate the causal relationships between immune cell-mediated processes and ESCC. We identified a genetic predisposition for naïve T-cell infiltration and its causal relationship with ESCC, leveraging MR. Additionally, the single-cell sequencing results indicate that naïve T-cells, under stress conditions, exhibit a diminished capacity to differentiate into effector T-cells, potentially reinforcing tumor evasion mechanisms. This groundbreaking approach sheds light on the intricate genetic factors influencing immune cell infiltration in ESCC and provides insights into the functional alterations of naïve T-cells within the tumor microenvironment. The identification of causal relationships and stress-induced functional changes in naïve T-cells enriches our understanding of the complex interplay between the immune system and ESCC progression, offering potential avenues for targeted interventions in cancer immunotherapy. The insights from our study into the role of naïve T-cells in ESCC offer significant clinical implications. By identifying a causal relationship between naïve T-cell phenotypes and ESCC, as well as revealing their altered states within the tumor microenvironment, our findings highlight potential new avenues for therapeutic intervention. Specifically, targeting the stress-induced alterations and metabolic reprogramming of naïve T-cells could lead to novel strategies for enhancing immune responses and improving patient outcomes. Additionally, these results underscore the importance of considering immune cell dynamics in the development of personalized treatment approaches and provide a foundation for future research aimed at refining immunotherapy strategies for ESCC.

Through single-cell sequencing, we delved deeper into unraveling the potential mechanisms behind the causal relationship between naïve T-cells and ESCC. Our findings revealed that naïve T-cells in tumor tissues, compared with their counterparts in normal tissues, exhibit reduced infiltration and maturation states. They display elevated expression of heat shock-related proteins, such as HSPD1, HSPE1, HSPA1A, HSPA6, and DNAJB1, indicative of a stress-induced state. Previous studies have highlighted the significance of heat shock-related proteins in modulating immune responses^35^ and their potential roles in tumorigenesis.^36,37^ For instance, HSPD1 and HSPE1 have been implicated in regulating immune cell function and tumor progression.^38–41^ Additionally, elevated expression of HSPA1A and HSPA6 has been associated with increased tumor aggressiveness and poor prognosis in various cancer types.^42–45^ Moreover, DNAJB1 has been reported to play a role in immune regulation and cancer development.^46,47^

Furthermore, the altered expression of these heat shock-related proteins in naïve T-cells within the tumor microenvironment underscores the complex interplay between stress responses and immune cell dynamics in cancer progression. It suggests that the stress-induced state of naïve T-cells may contribute to immune dysfunction and tumor immune evasion mechanisms. Additionally, naïve T-cells in tumor tissues demonstrate weakened capabilities in T-cell activation and differentiation processes. This is reflected in their diminished capacity for metabolic activities, as evidenced by lower expression levels in various metabolic pathways, including oxidative phosphorylation, glycolysis/gluconeogenesis, fatty acid degradation, cysteine and methionine metabolism, and the citrate cycle. These metabolic pathways play crucial roles in regulating T-cell function and activation. Oxidative phosphorylation provides energy for T-cell activation and effector function, whereas glycolysis supports rapid proliferation and cytokine production.^48–50^ Fatty acid degradation is essential for memory T-cell formation and long-term immune responses.^51,52^ Cysteine and methionine metabolism contribute to redox balance and immune regulation within the tumor microenvironment.^53^ The citrate cycle fuels T-cell metabolism and biosynthesis, supporting their survival and function in diverse microenvironments.^54,55^

The dysregulation of these metabolic pathways in naïve T-cells within the tumor microenvironment suggests a metabolic reprogramming that favors immune suppression and tumor growth. It highlights the intricate connection between cellular metabolism and immune function in cancer progression. Furthermore, through WGCNA, we identified potential regulatory genes associated with naïve -cells in tumor tissues. Two gene modules were found to be highly expressed in naïve T-cells within the tumor microenvironment. Subsequent visualization of these modules revealed the presence of various HSP-related genes. This further substantiates the notion that naïve T-cells in tumor tissues are under a state of stress.^56,57^ The identification of these gene modules not only highlights potential key regulators influencing naïve T-cell behavior in the tumor microenvironment but also provides a molecular framework for understanding the mechanisms underlying their altered function. The visualization of HSP-related genes within these modules adds another layer of evidence, reinforcing the hypothesis that stress-related pathways play a crucial role in shaping the phenotype and function of naïve T-cells in the context of ESCC. Moreover, Tregs have emerged as critical players in tumor development and immune-evasion mechanisms.^58–60^ Tregs exert suppressive effects on anti-tumor immune responses and facilitate immune tolerance within the tumor microenvironment.^60^ Importantly, studies have shown that naïve T-cells have the potential to differentiate into Tregs under certain conditions, contributing to the establishment of an immunosuppressive tumor microenvironment.^61,62^ This process of naïve T-cell differentiation into Tregs further underscores the dynamic interplay between different T-cell subsets and their impact on tumor progression.

This study offers several strengths that enhance the validity and depth of our findings. First, the comprehensive approach combining MR and single-cell transcriptome sequencing provides a robust framework for exploring causal relationships between immune cell phenotypes and ESCC. This integrative methodology allows for a more thorough investigation of the underlying mechanisms. Additionally, the study’s rigorous validation process, which included multiple statistical methods such as IVW, WM, inverse variance weighted, and weighted mode, ensures the reliability of our results. Sensitivity analyses, along with tests for heterogeneity and pleiotropy, further strengthen the robustness of our findings. Moreover, the detailed single-cell transcriptome analysis offers high-resolution insights into the immune cell landscape within tumor and normal tissues. Specifically, the in-depth analysis of T-cell subtypes and their functional states provides valuable information on the microenvironmental changes associated with ESCC. Furthermore, the identification of key molecular pathways, such as significant alterations in stress response and metabolic pathways in naïve T-cells, contributes to potential therapeutic strategies targeting the tumor microenvironment and immune cell functionality.

However, the study also has several limitations. The generalizability of the findings is constrained by the single-cell transcriptome analysis being performed on a limited number of patient samples. A larger and more diverse cohort would be necessary to ensure that the results are applicable across different populations and disease stages. Additionally, while several variables were controlled for, there remains the possibility of residual confounding from unmeasured factors. Future studies incorporating additional covariates and exploring potential confounding factors are needed to address this issue. The focus on specific T-cell subtypes, particularly naïve T-cells, while providing detailed insights, leaves other immune cell types and their interactions with naïve T-cells less explored. Expanding the analysis to include a broader range of immune cell networks could offer a more comprehensive understanding of ESCC. Lastly, the single-cell transcriptome analysis is subject to technological limitations, such as potential dropout events and sequencing depth. Advances in technology and methodology could improve the resolution and accuracy of future analyses.

The findings of our study suggest several promising directions for future research. One key area is the need for mechanistic studies to elucidate how stress and metabolic alterations affect naïve T-cell function and contribute to ESCC progression. Investigating the molecular pathways and cellular interactions involved could yield deeper insights into the observed functional changes and identify specific targets for therapeutic intervention. Additionally, our results point to the potential for developing targeted therapies that modulate stress responses and metabolic shifts in naïve T-cells. Such strategies could enhance immune function and improve treatment outcomes for patients with ESCC. Translating these findings into clinical practice will require further validation through clinical trials. Specifically, investigating naïve T-cell markers as diagnostic or prognostic biomarkers could provide new tools for monitoring disease progression and therapeutic response. Furthermore, clinical trials aimed at modifying naïve T-cell behavior could evaluate the effectiveness of these interventions in improving patient outcomes. Expanding the scope of research to include other cancer types could also help determine whether the stress-induced states and metabolic changes in naïve T-cells are unique to ESCC or represent a broader phenomenon across different cancers. Comparative studies could reveal common pathways and identify universal therapeutic targets. Finally, longitudinal studies tracking naïve T-cells throughout cancer progression and treatment could offer valuable insights into their evolution and role in disease dynamics. These studies could help refine therapeutic strategies and enhance our understanding of immune cell behavior in cancer. By pursuing these research directions, we can further elucidate the role of naïve T-cells in cancer and potentially develop innovative approaches for improving cancer treatment and patient care.

In summary, this study provides significant insights into the role of naïve T-cells in ESCC and identifies key areas for further investigation. Addressing these limitations in future research will help to refine our understanding of the complex interactions between immune cells and cancer.

## Supplementary Information

Below is the link to the electronic supplementary material.

Supplementary file1 (DOCX 18 KB)

## Data Availability

The original datasets analyzed in this study are publicly available through the following repositories: the GWAS Catalog (https://www.ebi.ac.uk/gwas/) and NCBI Gene Expression Omnibus (https://www.ncbi.nlm.nih.gov/gds/). Further inquiries can be directed to the corresponding authors.
