# Supplementary material for: Exploring the Link Between Stress-Induced Naive T-Cells and Esophageal Squamous Cell Carcinoma Risk: A Multi-Omics Investigation
Source: Ann Surg Oncol. 2025 Nov 4;33(2):1789–805. doi: 10.1245/s10434-025-18541-w (PMC12765749; doi:10.1245/s10434-025-18541-w)
Supplement: Supplementary file 1 — Supplementary file1 (DOCX 18 KB) [file 10434_2025_18541_MOESM1_ESM.docx]

Table S1: The results of reverse Mendelian randomization analysis.

|  | id.exposure | id.outcome | outcome | exposure | method | nsnp | b | se | pval | lo_ci | up_ci | or | or_lci95 | or_uci95 |
| --- | --- | --- | --- | --- | --- | --- | --- | --- | --- | --- | --- | --- | --- | --- |
| 1 | ebi-a-GCST90018841 | ebi-a-GCST90001566 | Naive CD4-CD8- T cell Absolute Count \|\| id:ebi-a-GCST90001566 | \|\| id:ebi-a-GCST90018841 | MR Egger | 68 | 0.030615 | 0.023419 | 0.195658 | -0.01529 | 0.076516 | 1.031088 | 0.98483 | 1.079519 |
| 2 | ebi-a-GCST90018841 | ebi-a-GCST90001566 | Naive CD4-CD8- T cell Absolute Count \|\| id:ebi-a-GCST90001566 | \|\| id:ebi-a-GCST90018841 | Weighted median | 68 | -0.00375 | 0.021816 | 0.863673 | -0.04651 | 0.039014 | 0.996261 | 0.954559 | 1.039785 |
| 3 | ebi-a-GCST90018841 | ebi-a-GCST90001566 | Naive CD4-CD8- T cell Absolute Count \|\| id:ebi-a-GCST90001566 | \|\| id:ebi-a-GCST90018841 | Inverse variance weighted | 68 | 0.007227 | 0.013277 | 0.586192 | -0.0188 | 0.03325 | 1.007254 | 0.98138 | 1.033809 |
| 4 | ebi-a-GCST90018841 | ebi-a-GCST90001566 | Naive CD4-CD8- T cell Absolute Count \|\| id:ebi-a-GCST90001566 | \|\| id:ebi-a-GCST90018841 | Simple mode | 68 | -0.03102 | 0.039385 | 0.433772 | -0.10821 | 0.046179 | 0.969461 | 0.897439 | 1.047262 |
| 5 | ebi-a-GCST90018841 | ebi-a-GCST90001566 | Naive CD4-CD8- T cell Absolute Count \|\| id:ebi-a-GCST90001566 | \|\| id:ebi-a-GCST90018841 | Weighted mode | 68 | 0.002342 | 0.024045 | 0.922709 | -0.04479 | 0.049471 | 1.002344 | 0.956201 | 1.050715 |
